# Identification of the Roles of Chromobox Family Members in Gastric Cancer: A Study Based on Multiple Datasets

**DOI:** 10.1155/2020/5306509

**Published:** 2020-11-05

**Authors:** Zhuo-Yuan Chen, Shang-Xing Sun, Si-Xian Zhu, Jie Bu

**Affiliations:** ^1^The Second Xiangya Hospital, Central South University, 139th Renmin middle Road, Changsha, Hunan, China; ^2^Department of Surgery, Hanyang Hospital, Wuhan University of Science and Technology, No. 53, Moshuihu Road, Wuhan, Hubei, China; ^3^Department of Oncology, Tongji Hospital, Huazhong University of Science and Technology, 1095 JieFang Avenue, Wuhan, Hubei, China; ^4^Department of Orthopaedics, Hunan Cancer Hospital and The Affiliated Cancer Hospital of Xiangya School of Medicine, Central South University, 283 Tongzipo Road, Changsha, Hunan, China

## Abstract

**Background:**

As the important components in polycomb repressive complexes 1 (PRC1) and heterochromatin protein 1 (HP1), Chromobox (CBX) family members are involved in epigenetic regulatory function, transcriptional repression, and other cellular metabolisms. Increasing studies have indicated significant associations between CBX and tumorigenesis, which is a progression in different types of cancers. However, the information about the roles of each CBX in gastric cancer is extremely limited.

**Methods:**

We explored CBX mRNA expression, corrections with clinicopathological parameters, protein expression, prognostic values, enrichment analysis with several databases including Oncomine, Human Protein Atlas, UALCAN, Kaplan-Meier plotter, cBioPortal, GeneMANIA, and Enrichr.

**Results:**

In our study, comparing to the normal tissues, higher mRNA expression of CBX1/2/3/4/5/8 and lower mRNA expression of CBX7 were found in GC tissues while upregulations of CBX1/2/3/4/5/8 and downregulations of CBX7 were indicated to be significantly correlated to the nodal metastasis status and individual cancer stages in GC patients. As for protein level, the expression of CBX2/3/4/5/6 was higher and the expression of CBX7 was lower in the GC tissues than those in the normal. What is more, higher mRNA expression of CBX1/5/6/8 and lower mRNA expression of CBX7 were markedly correlated to poor outcomes of OS and FP in GC patients. Besides, high mutation rate of CBXs (42%) was observed in GC patients.

**Conclusions:**

We suggest that CBX5/7 may serve as potential therapeutic targets for GC while CBX1/8 may serve as potential prognostic indicators for GC.

## 1. Introduction

According to the Global Cancer Statistics 2018 report, gastric cancer (GC) ranks sixth among the most common malignant tumors worldwide [1], with close to 1.03 million new GC cases and 0.78 million cancer-related deaths in 2018. The GC incidence rates are much higher in the low/middle income countries with highest rates in Eastern Asia, Eastern Europe, and South America while they are much lower in the higher income countries [2–4]. Because a large number of patients are diagnosed with advanced GC at their initial diagnosis, the 5-year survival rate of the disease remains below 30% even with comprehensive treatment modalities [5]. Stomach adenocarcinoma (STAD) is the most common type of GC, which occurs in the gastric glandular cells and accounts for 95% of the total incidence of GC [6, 7]. Recently, researchers have made immense progress in understanding the mechanisms of occurrence, development, and metastasis in the disease. However, some of the molecular mechanisms of GC remain unclear. Early diagnosis can significantly improve the therapeutic outcome of GC. However, due to the lack of effective methods for early diagnosis, many patients are diagnosed in the advanced stages or with distant metastasis, which significantly decrease the therapeutic outcome. Thus, identifying effective biomarkers and potential targets are extremely important for the early detection and treatment of GC.

Eight members of the Chromobox (CBX) family of proteins have been identified in mammalian cells thus far, including the polycomb (CBX2/4/6/7/8) and heterochromatin protein 1 (HP1) (CBX1/3/5, also known as HP1*β*/*γ*/*α*) [8–10]. All of them contain a single N-terminal chromodomain [8]. The polycomb-related CBX proteins interact with H3K27me3 by their chromodomains and help in the recruitment and stabilization of PRC1 to specific regions of the chromatin [11, 12], while the HP1-related proteins also have the terminal chromoshadow domain. Moreover, the CBX family of proteins is reported to be involved in transcriptional repression, cell cycle regulation, tumor initiation, progression, development, and chromatin [9, 13]. Increasing evidence suggests that CBX family of proteins plays vital roles in various cancers. CBX5 was demonstrated to serve as an oncogenic role in GC and be targeted by microRNA-758-3p in the proliferation, migration, and invasion of GC [14]. CBX7 was overexpressed both in GC cell lines and GC tissues, and its overexpression was correlated with patient's age, clinical stage, and lymph node metastasis [15]. However, the precise functions of the different CBXs in the development and progression of GC remain unclear. Using several large public databases, we analyzed the expression and mutations in the different CBX family members in STAD patients to determine their expression levels, potential functions, and prognostic values in GC.

### 1.1. Method Oncomine

Oncomine (https://www.oncomine.org) is a publicly accessible online cancer database, containing gene expression array data [16, 17]. In our study, Oncomine was used to analyze the mRNA expression level of the CBX members in different cancers. We compared the mRNA expression of CBX members in cancer and normal tissues using Student's *t*-test after set the thresholds as follows: *P* value: 0.001 and fold − change : 1.5.

### 1.2. UALCAN

An interactive online public platform, UALCAN (http://ualcan.path.uab.edu/), can be used to estimate gene expression and correlation, methylation, and survival analysis [18] based on clinical data and level 3 RNA-seq data from TCGA database. In our study, we used UALCAN to analyze the expression of CBX family members, nodal metastasis status, and individual cancer stage in STAD patients based on mRNA levels with 34 normal and 415 STAD tissues. We compared the mRNA expression using Student's *t*-test with *P* < 0.05 considered as statically significant. The nodal metastasis status criterion of UALCAN was as follows: no regional lymph node metastasis was considered as N0, and metastases in 1 to 3, 4 to 9, and 10 or more axillary lymph nodes were considered to be N1, N2, and N3, respectively.

### 1.3. Human Protein Atlas

The Human Protein Atlas (https://www.proteinatlas.org) is a public database, which can be used to analyze mRNA and protein expression data and survival information of patients for nearly 20 common kinds of cancers [19]. In our study, we used this database to compare the protein expression level of different CBX members between normal and GC tissues using immunohistochemistry images.

### 1.4. Kaplan-Meier Plotter

Kaplan-Meier plotter (http://www.kmplot.com) is an interactive database to obtain mRNA and miRNA expression data and survival-related information for different cancers from the GEO, EGA, and TCGA databases [20–22]. In our study, we used the KM Plotter to explore the overall survival (OS) and first progression (FP) as the prognostic values for CBX members, according to the datasets (excluding GSE62254, based on KM Plotter suggestion) of GC having divided the 592 patient samples into a high and low group based on the median mRNA expression level. Moreover, we set the hazard ratio with 95% confidence intervals. A log rank *P* value <0.05 was considered statically significant difference.

### 1.5. cBioPortal

cBioPortal (http://www.cbioportal.org) is a publicly accessible tool which can be used to analyze and visualize different cancer genomics data [23, 24]. We selected the STAD (TCGA, provisional) dataset, including 369 cases with pathology reports, for the analysis of CBXs in cBioPortal. Then, we assessed the gene mutations, putative copy number alterations from GISTIC, and mRNA expression *z*-scores (RNA Seq V2 RSEM, *z*-score threshold ±2.0). Using cBioPortal, we analyzed the genetic alterations in the CBXs and their correlation with each other. For the correlation of CBXs with each other, Spearman′s correlation coefficient > 0.3 and *P* < 0.05 were considered to be significant.

### 1.6. GeneMANIA

GeneMANIA (http://www.genemania.org), an online system for network analysis, can be used for predicting and visualizing the protein-protein interaction (PPI) network and gene functional assays [25] and features several bioinformatics methods: physical interaction, gene coexpression, gene colocation, gene enrichment analysis, and website prediction. In our study, GeneMANIA was used to construct the gene networks and predict the functions of CBXs.

### 1.7. Enrichr Database

The online public database [26, 27], Enrichr (http://amp.pharm.mssm.edu/ Enrichr/), was used to obtain the data pertaining to Gene Ontology (GO) functional annotations and Reactome pathway enrichment analysis for CBXs. Finally, we used the R (ggplot2 package) to create plots and visualize the data from our study.

## 2. Results

### 2.1. mRNA Expression of CBXs in Different Cancers

**Figure 1:**
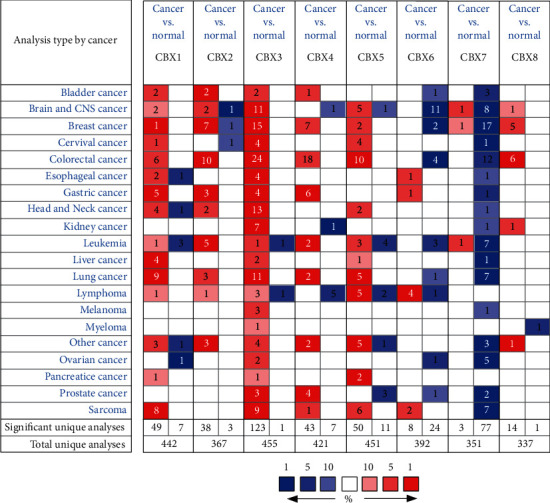
Transcriptional levels of the CBX family members in different cancers (Oncomine, *P* value: 0.001 and fold − change : 1.5).

We compared the mRNA expression of the CBXs in cancer and normal samples (Figure 1) using the Oncomine database and found that the transcriptional levels of CBX1/2//3/4/5/8 were upregulated while that of CBX6/7 were downregulated in majority of the cancers. In the GC samples (Table 1), CBX1 was markedly higher in GC patients in five datasets. In Cho's gastric dataset [28], CBX1 was overexpressed in gastric adenocarcinoma (fold − change = 2.415 and *P* = 4.52*E* − 6). In Chen's gastric dataset [29], CBX1 was found to be upregulated in diffuse gastric adenocarcinoma (fold − change = 1.516 and *P* = 2.83*E* − 08), gastric mixed adenocarcinoma (fold − change = 1.66 and *P* = 2.25*E* − 6), gastric intestinal-type adenocarcinoma (fold − change = 1.613 and *P* = 2.38*E* − 13). In D'Errico's gastric dataset [30], CBX1 was found to be upregulated in gastric intestinal-type adenocarcinoma (fold − change = 2.116 and *P* = 2.21*E* − 13). The mRNA expression of CBX2 was higher in GC patients in three datasets. In Cho's gastric dataset [28], CBX2 was overexpressed in diffuse gastric adenocarcinoma (fold − change = 2.29 and *P* = 6.01*E* − 09) and gastric mixed adenocarcinoma (fold − change = 2.077 and *P* = 3.75*E* − 04). D'Errico's gastric dataset [30] showed CBX2 to be upregulated in gastric intestinal-type adenocarcinoma (fold − change = 4.485 and *P* = 1.7*E* − 09). The mRNA expression of CBX3 was higher in GC patients in four datasets. Chen's gastric dataset [29] revealed that CBX3 was overexpressed in gastric mixed adenocarcinoma (fold − change = 1.998 and *P* = 1.62*E* − 07) and gastric intestinal-type adenocarcinoma (fold − change = 1.878 and *P* = 1.13*E* − 16). Moreover, CBX3 was overexpressed in gastric intestinal-type adenocarcinoma (fold − change = 3.014 and *P* = 6.64*E* − 14) in D'Errico's gastric dataset [30] and GC (fold − change = 1.736 and *P* = 6.79*E* − 04) of Wang's gastric dataset [31]. The mRNA expression of CBX4 was higher in GC patients in six datasets. In Chen's gastric dataset, CBX4 was overexpressed in gastric intestinal-type adenocarcinoma (fold − change = 1.783 and *P* = 2.55*E* − 17), gastric mixed adenocarcinoma (fold − change = 1.955 and *P* = 3.03*E* − 06), and diffuse gastric adenocarcinoma (fold − change = 1.730 and *P* = 4.23*E* − 04). D'Errico's gastric dataset [30] showed that CBX4 was upregulated in diffuse gastric adenocarcinoma (fold − change = 2.466 and *P* = 2.45*E* − 05) and gastric mixed adenocarcinoma (fold − change = 3.314 and *P* = 2.29*E* − 06). Cho's gastric dataset [28] indicated that CBX4 was higher in gastric mixed adenocarcinoma (fold − change = 1.625 and *P* = 7.18*E* − 04). The mRNA expression of CBX6 was higher in GC patients in one dataset. Chen's gastric dataset [29] revealed that CBX6 was upregulated in diffuse gastric adenocarcinoma (fold − change = 1.758 and *P* = 8.38*E* − 05). The mRNA expression of CBX7 was lower in GC patients in one dataset. In Cho's gastric dataset [28], CBX7 was downregulated in diffuse gastric adenocarcinoma (fold − change = −1.656 and *P* = 9.09*E* − 05).

### 2.2. mRNA and Protein Expression of CBXs in GC and Normal Samples

**Figure 2:**
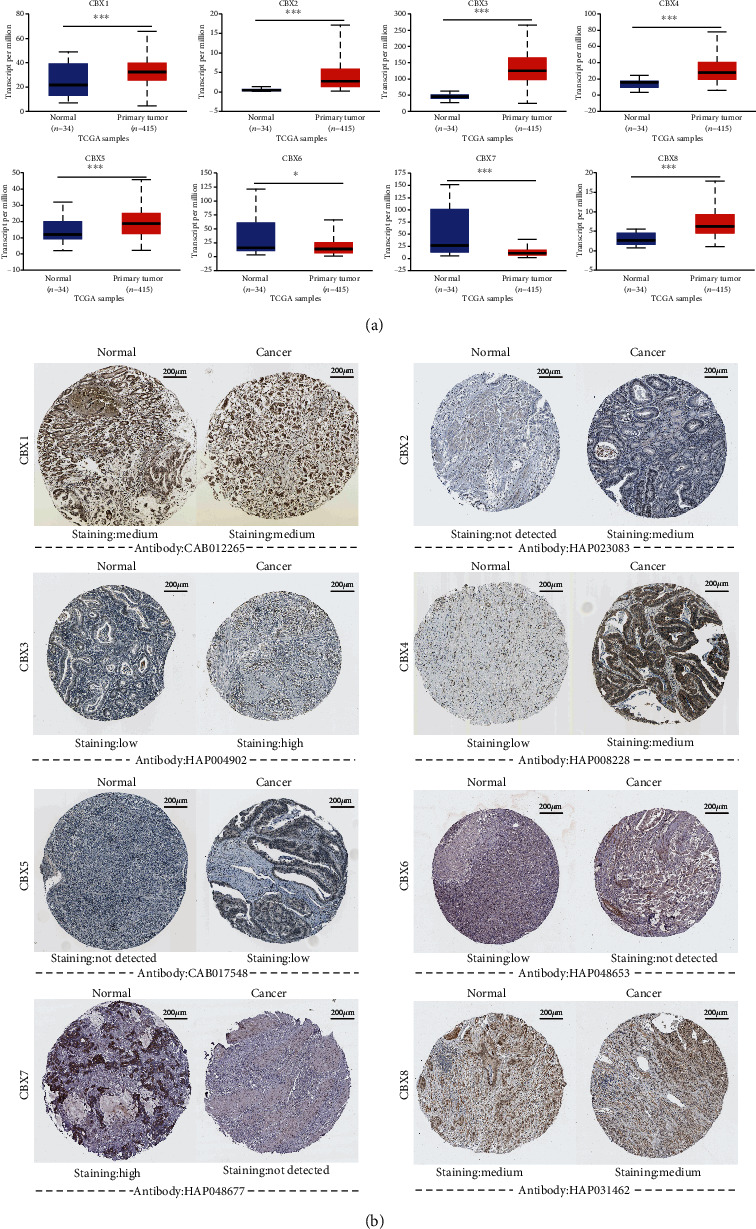
mRNA and protein expression of CBXs (UALCAN and Human Protein Atlas): (a) mRNA expression of different CBXs in STAD and normal tissues (UALCAN, ^∗^*P* < 0.05, ^∗∗^*P* < 0.01, and ^∗∗∗^*P* < 0.001); (b) protein expression of different CBXs in GC and the normal tissues (Human Protein Atlas).

The mRNA expression data from the UALCAN database revealed that the expression of CBX1/2/3/4/5/8 was significantly upregulated in the STAD tissues compared to the normal tissues, while the expression of CBX6/7 was markedly downregulated (Figure 2(a); please see the Supplementary 1 for primary data). Then, using the Human Protein Atlas, we evaluated the immunohistochemistry (IHC) data pertaining to the protein expression of CBXs in GC and normal tissues. We found that the protein expression of CBX2/3/4/5/6 was higher in GC tissues than normal tissues while that of CBX7 was lower in GC tissues than in the normal tissues (Figure 2(b)). The protein expression of CBX1/8 did not show much difference.

### 2.3. Clinicopathological Parameters of CBXs in GC Patients

**Figure 3:**
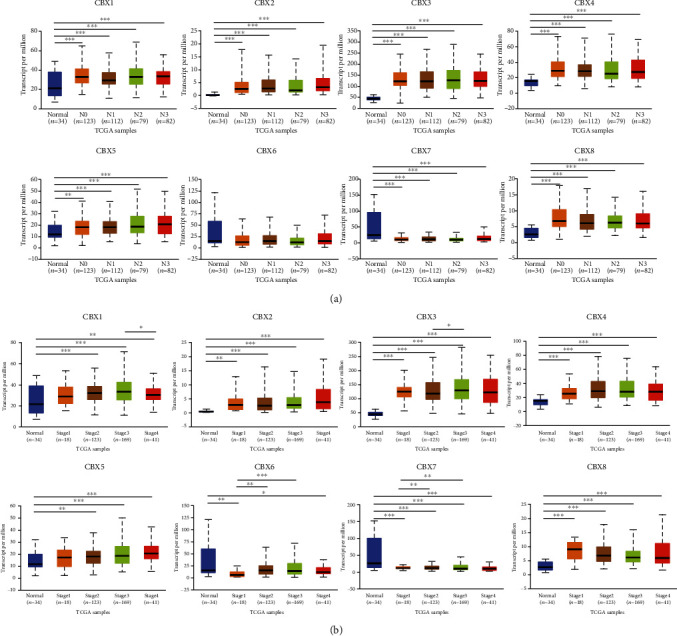
Clinicopathological parameters and CBX mRNA levels in STAD patients (UALCAN): (a) correlation between the expression level of CBXs and nodal metastatic status in STAD patients (UALCAN, ^∗^*P* < 0.05, ^∗∗^*P* < 0.01, and ^∗∗∗^*P* < 0.001); (b) correlation between expression of CBXs and individual cancer stage in STAD patients (UALCAN, ^∗^*P* < 0.05, ^∗∗^*P* < 0.01, and ^∗∗∗^*P* < 0.001).

Using the STAD database in UALCAN, we evaluated the correlation of CBX mRNA expression with the nodal metastatic status (Figure 3(a); please see the Supplementary 2 for primary data) and cancer stage of individual patients (Figure 3(b); please see the Supplementary 3 for primary data). As shown in Figure 3(a), the expression of CBX1/2/3/4/5/7/8 was all correlated with the nodal metastatic status while the expression of CBX6 had no correlation with the nodal metastatic status. Compared to normal tissues, the mRNA expression of CBX2/3/4/8 was significantly higher in the cancer stages 1, 2, 3, and 4, while the mRNA expression of CBX1 and CBX5 was significantly upregulated in the cancer stages 2, 3, and 4. Moreover, the mRNA expression of CBX7 was found to be downregulated in all cancer stages. The mRNA expression of CBX6 was lower in the cancer stages 1 and 4 compared to the normal tissues.

### 2.4. Prognostic Value of mRNA Level of CBXs in GC Patients

**Figure 4:**
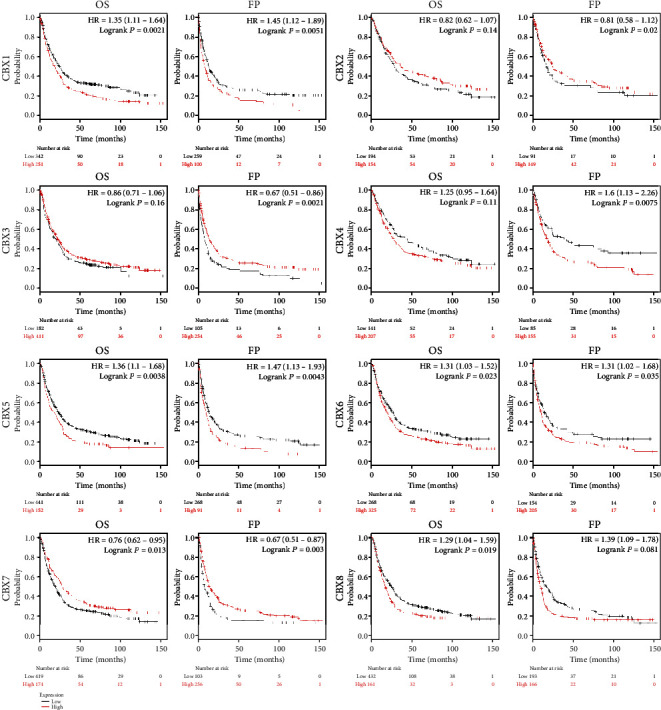
Prognostic value of mRNA level of CBX family members in GC patients (Kaplan-Meier plotter, *P* < 0.05 was considered statistically significant).

Using the KM Plotter, we explored the prognostic value of mRNA expression of CBXs according to the OS and FP of GC patients. The survival curve indicated that higher mRNA expression of CBX1/5/6/8 and lower mRNA expression of CBX7 predicted poor OS and FP (Figure 4).

### 2.5. Genetic Alterations and Interaction Analysis of CBXs in GC

**Figure 5:**
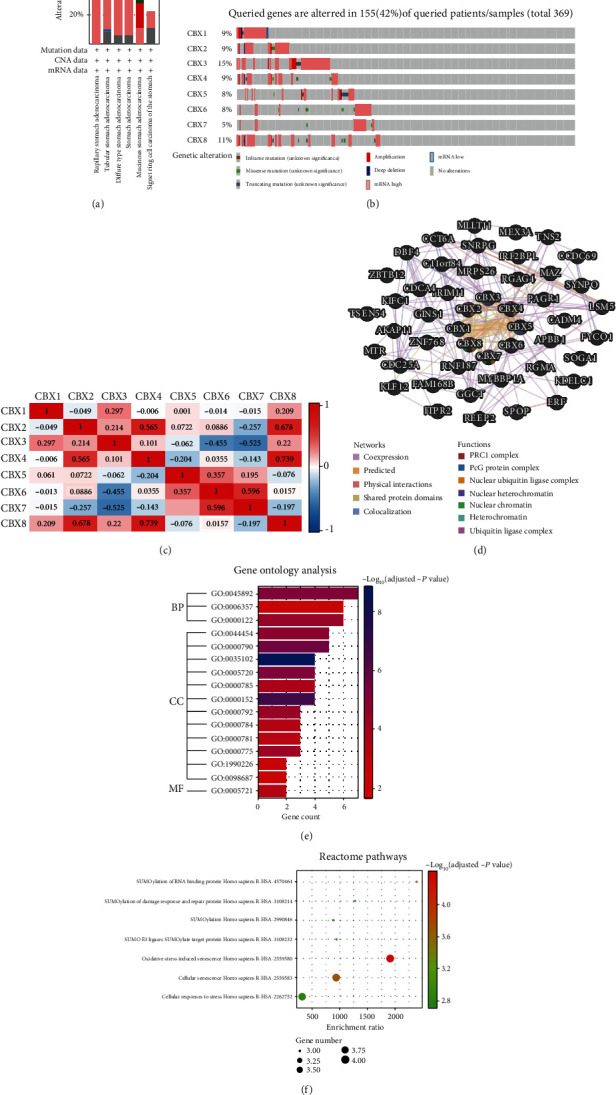
Genetic alterations, interactions, and enrichment analysis of CBXs in GC: (a) summary of CBX alteration in STAD; (b) alterations in CBXs in GC; (c) correction of CBXs with each other (cBioPortal); (d) interaction analysis of CBXs (GeneMANIA). Summary of alterations in CBXs in HCC: (e) GO enrichment analysis; (f) Reactome pathway prediction. BP: biological processes; CC: cellular components; MF: molecular functions.

Using cBioPortal, we analyzed the alterations in CBXs and their correlations with each other in STAD. The results showed that the CBXs were altered in 155 out of 369 patients with STAD (42%) (Figure 5(a)). Based on TCGA provisional dataset, the percentage of genetic alterations in CBX1/2/3/4/5/6/7/8 was 9, 9, 15, 9, 8, 8, 5, and 11%, respectively, in STAD (Figure 5(b)). Further, by analyzing the mRNA expression of the CBXs, we calculated the correlation of the CBXs with each other using Spearman's correlation (Figure 5(c); please see the Supplementary 4 for primary data). The results revealed significant positive correlations between in the following CBX pairs: CBX2 with CBX4 (*r* = 0.58 and *P* = 3.53*E* − 04); with CBX8 (*r* = 0.56 and *P* = 5.45*E* − 04); CBX4 with CBX8 (*r* = 0.62 and *P* = 9.63*E* − 05); CBX6 with CBX7 (*r* = 0.56 and *P* = 5.27*E* − 04); while significant negative correlations were found between the following pairs of CBXs; CBX2 with CBX7 (*r* = −0.54 and *P* = 8.88*E* − 04); CBX3 with CBX6 (*r* = −0.40 and *P* = 1.81*E* − 02).

The GeneMANIA (Figure 5(d); please see the Supplementary 5 for primary data) results revealed that the CBXs shared protein domains with each other. Interactions were predicted between CBX1 and CBX3, CBX1 and CBX5, CBX3 and CBX5, and CBX4 and CBX8. Moreover, CBX1 and CBX2, CBX1 and CBX3, CBX1 and CBX5, CBX2 and CBX4, CBX3 and CBX5, CBX4 and CBX6, CBX4 and CBX8, and CBX7 and CBX8 shared physical interactions. Relationships of coexpression were predicted between CBX1 and CBX8, CBX2 and CBX4, CBX3 and CBX5, and CBX6 and CBX7.

### 2.6. Enrichment Analysis of CBXs

To identify the potential signaling pathways of CBXs in GC, we used the Enrichr online database to analyze GO functional annotation (based on biological processes (BPs), cellular components (CCs), and molecular functions (MPs) and the Reactome pathways; please see the Supplementary 6 for primary data). According to the results (Figure 5(e)), CBXs were mainly involved in negative regulation of transcription, DNA templated (GO:0045892), negative regulation of transcription from RNA polymerase II promoter (GO:0000122), and regulation of transcription from RNA polymerase II promoter (GO:0006357) in BPs. As for CCs, the CBXs were mainly involved in PRC1 complex (GO:0035102), nuclear ubiquitin ligase complex (GO:0000152), nuclear heterochromatin (GO:0005720), heterochromatin (GO:0000792), and chromatin (GO:0000785). Moreover, CBXs influenced MFs through histone methyltransferase binding (GO:1990226).

In Reactome analysis (Figure 5(f)), 7 pathways were of significance, including oxidative stress-induced senescence Homo sapiens R-HSA-2559580, cellular senescence Homo sapiens R-HSA-2559583, SUMOylation of RNA binding protein Homo sapiens R-HSA4570464, SUMOylation of DNA damage response and repair protein Homo sapiens R-HSA-3108214, SUMO E3 ligases SUMOylate target protein Homo sapiens R-HSA3108232, SUMOylation Homo sapiens R-HSA-2990846, and cellular responses to stress Homo sapiens R-HSA-2262752.

## 3. Discussion

To date, some of CBXs have been reported to be involved in tumorigenesis and progression of several cancers, but the function of CBXs in GC is limited. This is the first study to systematically analyze their mRNA and protein expression, as well as evaluate their correlation with the clinicopathological parameters, prognostic values, genetic alterations, and potential functions in GC. We believe that our study will contribute toward improved early diagnosis, treatment outcome, and prognosis for patients with GC.

Increasing number of studies have shown that CBX1, also known as HP1-*β*, is overexpressed in different type of tumors, including prostate cancer (PRCA), breast cancer (BRCA), and hepatocellular carcinoma (HCC) [13, 32, 33]. Overexpression of CBX1 was associated with poor recurrence-free survival (RFS) in BRCA patients [13]. In addition, high expression of CBX1 was markedly correlated with larger tumor size, poor tumor differentiation, and tumor vascular invasion in hepatocellular carcinoma [33]. However, Tretiakova et al. [34] reported that the expression of CBX1 was lower in thyroid carcinoma, and the decrease in CBX1 followed by a reduction in CBX5 contributed to the pathogenesis of thyroid carcinoma. Our study indicated that CBX1 mRNA expression was upregulated in GC patients, which was in accordance with the data in other tumors. However, its protein expression did not show much difference between GC and normal tissues. Further, its mRNA expression was significantly correlated with nodal metastatic status, individual cancer stage, and poor OS and FP in GC patients. Taken together, our data indicate that CBX1 may serve as a potential prognostic marker in GC.

Recent studies have confirmed that CBX2 acts as an oncogene in several cancers, including BRCA, HCC, PRCA, and ovarian cancer (OVCA) [35–38]. Piqué et al. found that CBX2 promotes BRCA cell growth, and overexpression of CBX2 was related to poorer 5-year survival [37]. In HCC, Mao et al. confirmed that CBX2 was highly expressed both in HCC cell lines and tissues and was associated with poor prognosis in patients [36]. Clermont et al. revealed that elevated CBX2 expression was associated with poor clinical outcome in PRCA [35]. Our study indicated that mRNA and protein expression of CBX2 were significantly overexpressed in GC compared to normal tissues, while it was also correlated with nodal metastatic status and individual cancer stage in GC patients. However, lower CBX2 expression was associated with lower OS and PF in the patients with GC, but the difference was not significant.

CBX3 is highly expressed in lung adenocarcinoma (LUAD), pancreatic cancer (PACA), colorectal cancer (CRC), PRCA, non-small-cell lung cancer (NSCLC), and tongue squamous cell carcinoma (TSCC) [39–44]. Alam et al. found that CBX3 enhanced the expression of protumorigenic genes in LUAD by downregulating NCOR2 and ZBTB7A [39]. Chen et al. reported that CBX3 promoted aerobic glycolysis by suppressing FBP1 in PACA cell [42]. Liu et al. demonstrated that CBX3 was correlated with poor prognosis in CRC and promoted the proliferation and tumorigenesis of CRC, while miR-30a targeted CBX3 to specifically suppress the growth of CRC in a xenograft mouse model [43]. In our report, we confirmed that both mRNA and protein expression of CBX3 were upregulated in GC tissues compared to normal tissues. The mRNA expression was also significantly associated with nodal metastatic status and individual cancer stage. Lower mRNA expression of CBX3 was associated with poor FP in GC, but the OS result was not significant.

As an important member in the CBX family, CBX4 was found to promote several cancers, including HCC, lung cancer, and BRCA [45–47]. Li et al. revealed that the expression of CBX4 was markedly related to VEGF expression, angiogenesis, and OS in HCC and that CBX4 was important for tumor angiogenesis by governing HIF-1*α* protein [46]. In lung cancer, CBX4 regulated the expression of BMI-1 to promote proliferation and metastasis in lung cancer cells, and its expression was positively correlated with tumor size [45]. Surprisingly, Wang et al. indicated that CBX4 suppressed metastasis through recruitment of HDAC3 to the runx2 promoter in CRC [48]. In our study, the mRNA and protein expression of CBX4 were higher in GC tissues compared to normal tissues, and the mRNA expression was significantly correlated with nodal metastatic status and individual cancer stage in patients with GC. Moreover, higher CBX4 mRNA expression was correlated with worse OS and FP in GC, although the differences were not significant.

CBX5, also known as HP1*α*, was reported to be associated with several cancers, including GC, PRCA, BRCA, and NSCLC [14, 49–51]. A previous study showed that overexpression of CBX5 promoted cell proliferation in NSCLC cell lines [51]. Lieberthal et al. revealed that the expression of CBX5 was reduced in BRCA cell lines, while YY1 expression was detected to be lower in the invasive BRCA cell line [50]. In our report, both the mRNA and protein expression of CBX5 were upregulated in GC tissues than that in normal tissues. The mRNA expression was significantly correlated with nodal metastatic status and individual cancer stage in patients with GC. Moreover, its higher mRNA expression was significantly correlated with poor OS and FP. Thus, the results revealed that CBX5 may serve an oncogenic role in GC.

The mechanism of CBX6 is complex in different types of cancers. Zheng et al. found that higher CBX6 expression in HCC patients was associated more frequently with larger tumor sizes and multiple tumors [52]. Deng et al. revealed that CBX6 was downregulated in BRCA and negatively regulated by EZH2 [53]. In our study, the function of CBX6 was ambiguous. The mRNA and protein expression of CBX6 were significantly increased in GC tissues than normal tissues. However, in the STAD databases, the mRNA expression of CBX6 was significantly decreased, and the downregulation of the mRNA was associated with individual cancer stages 1 and 4 but of no significant correction with nodal metastatic status. Higher mRNA expression of CBX6 was significantly correlated with poor OS. Taken together, our results seem inconsistent with the predicted role of CBX6 as a gene of prognostic value, and further studies are needed to determine the precise function of CBX6 in GC in the future.

Among the CBXs, CBX7 is most studied in cancers. CBX7 was reported to be a tumor suppressor gene. The loss or downregulation of CBX7 gene expression was associated with several cancer, including PACA, thyroid cancer (THCA), CRC, NSCLC, bladder carcinoma (BLCA), and HCC [54–59]. Pallante et al. [56] reported that compared to the normal colonic mucosa, CBX7 expression was decreased or missing in a significant number of CRC samples. In THCA, the loss of CBX7 expression was correlated with larger tumor size in THCA patients while CBX7 expression progressively decreased with malignancy grade and neoplasia stage [55]. Moreover, Karamitopoulou et al. [54] showed that loss of CBX7 expression was related to increased tumor grade in PACA.

Surprisingly, Zhang et al. [15] found that CBX7 was overexpressed in GC cell lines and tumor tissues while its overexpression was also correlated to patient's age, lymph node metastasis, and clinical stage. Our study demonstrated that the mRNA and protein expression of CBX7 were markedly decreased in GC tissues compared to normal tissues. Moreover, the downregulation of CBX7 mRNA expression was markedly correlated with nodal metastatic status and individual cancer stage and poor OS and FP in GC patients. These results indicate that CBX7 may serve as a tumor suppressor in GC.

Recent studies show that CBX8 promotes tumorigenesis in several cancers, including BRCA, HCC, and CRC [60–62]. Overexpression of CBX8 in HCC patients was positively associated with distant metastasis and inversely correlated with OS. CBX8 promoted HCC cell proliferation capacity [61]. Yang et al. [62] revealed that silencing CBX8 induced apoptosis in CRC cell lines. Our report confirmed that the mRNA expression of CBX8 was higher in GC tissues than in normal tissues while its protein expression did not show much difference. Moreover, the mRNA expression was significantly correlated with nodal metastatic status and individual cancer stage in patients with GC. High CBX8 mRNA expression was associated with poor OS and FP in GC. These results indicate that CBX8 may of prognostic value in GC.

However, our study has some limitations. Firstly, the data in our study were from public databases, and further studies are needed to validate our results. Secondly, we did not explore the molecular mechanisms of different CBXs in GC, and future studies are needed to investigate their detailed mechanisms.

## 4. Conclusion

In conclusion, compared to normal tissues, higher mRNA expression of CBX1/2/3/4/5/8 and lower mRNA expression of CBX7 were found in GC, while the results for CBX6 were ambiguous. The protein levels of CBX2/3/4/5/6 were higher while that of CBX7 was lower in the GC tissues compared to normal tissues. The upregulation of the mRNA of CBX1/2/3/4/5/8 and downregulation of CBX7 were found to be significantly correlated to the nodal metastatic status and individual cancer stage in GC patients. Further, higher mRNA expression of CBX1/5/6/8 and lower mRNA expression of CBX7 were markedly associated with poor OS and FP in GC patients. High mutation rate of CBXs (42%) was observed in GC patients and to varying degrees. In summary, CBX5/7 may serve as a potential therapeutic target, while CBX1/8 may serve as potential prognostic factor in GC.

## Figures and Tables

**Table 1:** The transcription levels of CBX family members between different types of gastric cancers and normal gastric tissues (Oncomine).

	Types of gastric cancer vs. normal	Fold-change	*t*-test	*P* value	Ref	PMID
CBX1	Gastric adenocarcinoma vs. normal	2.415	6.913	4.52*E*-06	Cho gastric	21447720
	Diffuse gastric adenocarcinoma vs. normal	1.516	6.996	2.83*E*-08	Chen gastric	12925757
	Gastric mixed adenocarcinoma vs. normal	1.66	7.026	2.25*E*-06	Chen gastric	12925757
	Gastric intestinal-type adenocarcinoma vs. normal	1.613	9.531	2.38*E*-13	Chen gastric	12925757
	Gastric intestinal-type adenocarcinoma vs. normal	2.116	10.157	2.21*E*-13	D'Errico gastric	19081245
CBX2	Diffuse gastric adenocarcinoma vs. normal	2.29	6.862	6.01*E*-09	Cho gastric	21447720
	Gastric mixed adenocarcinoma vs. normal	2.077	4.349	3.75*E*-04	Cho gastric	21447720
	Gastric intestinal type adenocarcinoma vs. normal	4.485	7.31	1.7*E*-09	D'Errico gastric	19081245
CBX3	Gastric mixed adenocarcinoma vs. normal	1.998	8.875	1.62*E*-07	Chen gastric	1292575
	Gastric intestinal-type adenocarcinoma vs. normal	1.878	11.061	1.13*E*-16	Chen gastric	12925757
	Gastric intestinal-type adenocarcinoma vs. normal	3.014	9.795	6.64*E*-14	D'Errico gastric	19081245
	Gastric cancer vs. normal	1.736	3.719	6.79*E*-04	Wang gastric	21132402
CBX4	Gastric intestinal-type adenocarcinoma vs. normal	1.783	10.753	2.55*E*-17	Chen gastric	12925757
	Gastric mixed adenocarcinoma vs. normal	1.955	8.08	3.03*E*-06	Chen gastric	12925757
	Diffuse gastric adenocarcinoma vs. normal	1.73	4.295	4.23*E*-04	Chen gastric	12925757
	Diffuse gastric adenocarcinoma vs. normal	2.466	4.862	2.45*E*-05	D'Errico gastric	19081245
	Gastric mixed adenocarcinoma vs. normal	3.314	6.444	2.29*E*-06	D'Errico gastric	19081245
	Gastric mixed adenocarcinoma vs. normal	1.625	3.633	7.18*E*-04	Cho gastric	21447720
CBX5	NA	NA	NA	NA	NA	NA
CBX6	Diffuse gastric adenocarcinoma vs. normal	1.758	4.643	8.38E-05	Chen gastric	12925757
CBX7	Diffuse gastric adenocarcinoma vs. normal	-1.656	-4.072	9.09*E*-05	Cho gastric	21447720
CBX8	NA	NA	NA	NA	NA	NA

## Data Availability

All the data of this article is derived from public database in methods.

## Abbreviations

CBXs: Chromobox family members
GC: Gastric cancer
STAD: Stomach adenocarcinoma
GISTC: Genomic Identification of Significant Targets in Cancer
PPI: Protein-protein interaction
PcG: Polycomb group
HCC: Hepatocellular carcinoma
BRCA: Breast cancer
PRCA: Prostate cancer
LUAD: Lung adenocarcinoma
CRC: Colorectal Cancer
PACA: Pancreatic cancer
NSCLC: Non-small-cell lung cancer
THCA: Thyroid cancer
BLCA: Bladder carcinoma
CECA: Cervical cancer
OS: Over survival
FP: First progression
RFS: Recurrence-free survival
TCGA: The Cancer Genome Atlas
GO: Gene Ontology
BP: Biological processes
CC: Cellular components
MF: Molecular functions.
